# Erythema Nodosum in Children: A Narrative Review and a Practical Approach

**DOI:** 10.3390/children9040511

**Published:** 2022-04-04

**Authors:** Sandra Trapani, Chiara Rubino, Lorenzo Lodi, Massimo Resti, Giuseppe Indolfi

**Affiliations:** 1 Pediatric Unit, Department of Health Sciences, Meyer Children’s University Hospital, University of Florence, Viale Pieraccini 24, 50139 Florence, Italy; 2 Pediatric Unit, Meyer Children’s University Hospital, Viale Pieraccini 24, 50139 Florence, Italy; chiara.rubino@meyer.it (C.R.); massimo.resti@meyer.it (M.R.); 3 Immunology and Molecular Microbiology Unit, Meyer Children’s University Hospital, Viale Pieraccini 24, 50139 Florence, Italy; lorenzo.lodi@unifi.it; 4 Pediatric Unit, Department of NEUROFARBA, Meyer Children’s University Hospital University of Florence, Viale Pieraccini 24, 50137 Florence, Italy; giuseppe.indolfi@unifi.it

**Keywords:** erythema nodosum, children, work-up

## Abstract

Erythema nodosum (EN) is the most frequent form of panniculitis in children. We performed a literature review analyzing studies on pediatric EN published from 1990 to February 2022. EN is rare in pediatric age. It can be primary/idiopathic in 23–55% cases, or secondary in 47–77% cases. Secondary EN is related to a wide variety of conditions including infectious diseases, autoimmune disorders, malignancy, drugs, vaccinations, and pregnancy. The diagnosis of EN is clinical, based on the acute appearance of painful and red nodules localized to lower limbs, bilaterally distributed. If EN is diagnosed, basic work-up should include inflammatory markers, serum aminotransferases, lactate dehydrogenase, creatinine, protein electrophoresis, immunoglobulins, testing for streptococcal infection, and a tuberculin skin test. Based on the medical history and associated manifestations, further laboratory and radiological exams should be performed. The prognosis of EN is excellent, with spontaneous resolution in most patients within 2–6 weeks. Treatment, if needed, is addressed to the underlying condition. Despite being a rare manifestation in children, EN can be isolated or the first manifestation of a systemic or infectious condition. EN diagnosis is clinical, and a high index of suspicion is needed to perform investigations for the underlying disorders.

## 1. Introduction

Erythema nodosum (EN) is an uncommon pediatric skin disorder. It is, however, the most frequent form of panniculitis in children. The term panniculitis includes a heterogeneous group of inflammatory processes of subcutaneous adipose tissue classifiable in two categories, lobular and septal, according to histopathological criteria. The septal panniculitis is mainly represented by EN [1,2]. EN is an acute, usually self-limited, condition clinically characterized by the appearance of palpable subcutaneous nodules, typically painful, red or purplish in color, most often on the legs. It may be isolated or the first manifestation of systemic, autoimmune diseases, or malignancy [3]. Pediatricians are not always familiar with EN, and a high index of suspicion is needed to make a specific diagnosis for the related disorders, which can be rare and severe conditions. The purpose of this review is to provide an update of the etiopathogenetic features of EN in children, to focus on its clinical aspects, and define a practical approach and diagnostic pathway.

## 2. Materials and Methods

We analyzed the current literature of the triggering conditions of EN with a special focus on children. We used Embase^®^, MEDLINE^®^, MEDLINE^®^-In Process for English-language studies published from 1990 to February 2022. PubMed databases were searched combining the keywords “erythema nodosum” and “child” or “children” or “pediatric” and any of the following: “streptococcal infection, tuberculosis, Behçet disease, sarcoidosis, inflammatory bowel disease, drugs, vaccines, COVID-19, malignancies or cancer”, “epidemiology”, “diagnosis”. Titles and abstracts in English were evaluated for eligibility. References of selected relevant articles were reviewed and papers from these sources were also included.

## 3. Results

### 3.1. Epidemiology

The estimated prevalence of EN in the world is 1–5 per 100,000, varying upon the different geographic areas and the various associated triggering diseases [2]. A higher incidence (12–14 cases/100,000) was reported in Scandinavia [4]. It can manifest in all racial groups and ages, being more common between 18 and 40 years; in contrast, it is rare in pediatric age and exceptional in children before the age of 2 years [2,5]. In adults, the females–males ratio is 3–5:1 [1,6]. In children under 12 years, there is no prevalence of gender [5,7], while in adolescence, females are prevalent [7]. The female prevalence suggests that estrogen may be involved in the pathogenesis [7]. Furthermore, familiarity has been described. EN has been described as manifesting itself in several members with the same HLA [8].

### 3.2. Etiology

EN can be distinguished between a primary form, also called idiopathic, which represents a quote variable among 23% [7] up to 55% [3], and a secondary one ranging from 47% [6] to 77% [7]. Some authors found a lower rate of idiopathic EN in adults, as Porges et al. who reported 9% [4] and Garcia-Porrua et al. who reported 37% [9]. The secondary EN is related to a wide variety of conditions including infectious diseases (bacterial, viral, fungal, or protozoal), autoimmune disorders, malignancy, drugs, vaccinations, and pregnancy. In Table 1 are summarized the main EN-related etiologies considering all the pediatric studies, both series and single case reports. In Table 2 are summarized the etiologic factors reported from the pediatric case series published in the literature [5,7,10,11,12,13,14,15]; unfortunately, the pediatric case series are few and relatively small, especially compared to those of adults [6].

#### 3.2.1. Infectious Diseases

The most common infections, especially in childhood, are represented by Group A β-hemolytic Streptococcus (GAS), Mycobacterium, Yersinia, atypical mycobacteria, and Salmonella. All the numerous infectious agents responsible for EN are listed in Table 3. Streptococcal infections account for up to 44% of cases in adults [6]. In children, it has been variably reported ranging from 22% [5] to 48% of cases [7]; in these cases, EN may appear 2–3 weeks after an episode of streptococcal pharyngitis. Therefore, children with EN secondary to GAS infection do not necessarily present pharyngitis along with the skin lesions, but they certainly have a history of sore throat. Besides GAS, Mycoplasma pneumoniae represents a frequent infectious trigger for EN. In children, several studies described M. pneumoniae infection: Kakourou et al. found such recent infection in 3/35 cases (8.6%) [7]; Greco et al. reported two children with EN associated with M. pneumoniae infection, which occurred without pneumonia in one [17]; Shimuzu et al. reported an 8-year-old girl, who had EN followed by Henoch-Schönlein purpura, without pulmonary manifestations but with serology consistent with recent M. pneumoniae infection [18]. In the last study performed by Aydin-Teke, 2 out of 39 (5.1%) children with EN were infected with M. pneumoniae [11]. In the past, tuberculosis (TB) was the foremost cause of EN; recently, this association has decreased dramatically but has not disappeared [19]; in countries such as India, Turkey, and South Africa it remains an important cause. However, also in western countries, despite its low frequency, EN may be the first presentation of TB also in children and may be associated with latent TB infection or TB disease [20,21]. Notably, when a patient with TB presents at onset arthritis-like symptoms and EN, this is called Poncet’s disease, a rare presentation of TB [22]. Yersinia enterocolitica and pseudotuberculosis are important causes of EN in children with gastrointestinal diseases [5]. Mantakadis et al. described the case of an 8-year-old boy developing EN during the course of a febrile gastroenteritis caused by Salmonella enteritidis [23]. A recent report described the first two pediatric cases of EN as clinical presentation of melioidosis, a disease caused by Burkholderia pseudomallei, a Gram-negative bacillus which is endemic in tropical regions [24]. As shown in Table 3, besides the well-known viruses such as Epstein–Barr virus (EBV), cytomegalovirus (CMV), hepatitis B and C viruses, and parvovirus B19, also severe acute respiratory syndrome coronavirus-2 (SARS-CoV-2) has been recently added as a trigger of EN [25,26]. As regards systemic mycoses, the geographic area has a great value; for instance, in western/southwestern areas of the United States, EN is commonly caused by coccidioidomycosis [27]. Among fungal infections, kerion celsi, due to Trichophyton mentagrophytes, has been rarely reported [28]. In a retrospective study on 24 Israeli children, most cases were associated with streptococcal and EBV infection or with chronic inflammatory conditions, especially inflammatory bowel disease (IBD), but tuberculosis was not identified as a possible cause of the increase of its morbidity in that country [14].

#### 3.2.2. Systemic Diseases

Several systemic diseases are related to EN accounting for most cases in adults but less often implicated in the pediatric age. Among the granulomatous disorders, sarcoidosis has been frequently associated with EN (10–25%), particularly in young women [29,30]. Sarcoidosis with EN, hilar adenopathy, and polyarthritis is called Löfgren’s syndrome, an acute and self-limiting disease, that resolves in 6–8 weeks [31]. This phenomenon is rarely described in the pediatric population, although recently, the case of a 17-year-old boy with Löfgren syndrome has been reported [32]. Moreover, EN occurs in many autoimmune disorders such as IBD; in detail, EN has been described in 4–15% of patients with Crohn’s disease (CD) [28,33], and 3–10% of cases with ulcerative colitis (UC) [34]. The occurrence of EN with abdominal pain and diarrhea may reflect acute flare-ups of IBD [35,36]. Dotson et al. in a large study on pediatric IBD, found EN in 2.8% of the whole population (3.6% in CD patients, and 0.71% in UC) and confirmed the association between increased disease severity and EN occurrence [37]. Behçet disease (BD) [38], celiac disease [39,40], Sjogren syndrome [41], and spondyloarthropathy [42] are other autoimmune conditions associated with EN with varying prevalence. BD is the most frequently associated not only in adults (44%) [16] but also in children, where it accounts for 13.6–18.7% of cases in different studies [43,44]. Notably, EN can be the initial manifestation of the disease. Concerning CD, it was proposed that the augmented intestinal permeability to various antigens may provoke the skin hypersensitivity reaction; in the literature, it has been reported that CD may coexist with sarcoidosis which is a common cause of EN. EN associated with CD may be far more common than expected [45]. More rarely, autoimmune hepatitis [46], Takayasu arteritis [47], and Kawasaki Disease [48] have also been described as case reports in children. EN has been viewed as a cutaneous marker for several monogenic auto-inflammatory diseases such as Blau Syndrome [49], HIDS (Hyper-IgD syndrome) [50], and cryopyrin-associated periodic syndrome (CAPS) [51]. Furthermore, reports of concurrent Sweet’s Syndrome and EN have been published, and possible pathogenetic associations between these two reactive dermatoses have been supposed [52].

#### 3.2.3. Malignancy

A further group of EN-related diseases is represented by malignancies, particularly Hodgkin and non-Hodgkin lymphoma [53] and leukemia [51,52,53], even in pediatric age [54,55,56]; notably, it has been reported that EN development in a patient with a history of Hodgkin’s disease may reflect a recurrence of the disease [53]. In addition, solid tumors such as sarcoma, pelvic carcinoma, carcinoid tumor, renal, cervix, gastric, colorectal, pulmonary, hepatocellular, and pancreatic carcinoma have been reported in adults [57,58].

#### 3.2.4. Others

Several drugs, such as antibiotics, aspirin, oral contraceptives, azathioprine, phenytoin, valproate, and iodides, have been occasionally associated with EN, although less often in children than in adults. In children, the most common EN-related drugs have been penicillin, macrolides, and cephalosporin [59]. Proton pump inhibitors and leukotriene modifiers also have been implicated, although evidence is limited to a few case reports in adults [3,60]. Recently, everolimus treatment has been associated with EN arising in a 13-year-old girl with tuberous sclerosis complex [61]. The new onset of EN after receiving a vaccine is extremely rare but it has been reported following Bacillus-Calmette-Guerin, typhoid vaccine, cholera vaccine, human papilloma virus, and combined tetanus-diphteria- acellular pertussis vaccine [2,52]. In the last year, a few cases of EN associated with COVID-19 vaccines have been described, either with Pfizer [62], Moderna [63], and Astra Zeneca [64]. Pregnancy-associated EN, described in 2% of women with EN, has never been reported in adolescents with EN.

### 3.3. Pathophysiology and Histopathology

The pathogenesis of EN is not fully understood, although several authors consider EN a delayed-type hypersensitivity reaction due to exposure to different antigens (both internal and external). The mechanism may involve immune complex deposition in the septal venules of the subcutaneous fat [1,3,65]. EN is, in fact, the prototype of septal panniculitis without primary vasculitis: septa of subcutaneous adipose tissue appear thickened and infiltrated by inflammatory cells. Early lesions demonstrate predominant septal edema with prevalent neutrophils and eosinophils infiltrate, resulting in the reactive oxygen species formation; this is followed by a mild infiltrate of activated T lymphocytes CD4 with tumor necrosis factor (TNF) α production. Subsequently, a shift to an infiltrate of lymphocytes, macrophages, histiocytes, and multinucleated giant cells occurs; they aggregate themselves surrounding a cleft-like virtual space or small blood vessels, determining the so-called “Miescher radial granuloma” which is the pathognomonic EN feature [65,66]. Sometimes, inflammation can extend to the peri-septal areas of the adipose lobules [1,65]. The hypodermal, subcutaneous inflammation determine the nodule formation, whereas the inflammation of the dermal layer is responsible for the cutaneous redness. Lesions that are virtually identical to those of EN can be seen after the administration of biological therapeutic agents, as adalimumab or BRAF inhibitors [67]. High levels of interleukins (IL) (IL-6, IL-8, IL-12) and growth factors (TNF-α, interferon-γ, granulocyte colony-stimulating factor and monocyte chemoattractant protein-1) mainly involved in neutrophil recruitment and activation, have been reported in the skin and serum of EN patients [68].

### 3.4. Clinical Presentation

The sudden appearance of nodules may be preceded by a prodrome of fatigue, low-grade fever, malaise, joint pain, or upper respiratory tract infection (URTI) symptoms. The painful nodules are usually round or oval, warm, and covered by erythematous or violaceus skin; they are symmetrically distributed and commonly localized in the extensor surface of the legs (about 98%), although forearms, thighs, and trunk also may be affected [1,2,7]. Less frequently, lesions can coalesce or arise in unusual areas such as buttocks, calves, or face. They vary from 1 to 5 cm in diameter and are poorly demarcated, reflecting their subcutaneous anatomic location. Initially, nodules can be firm, but they usually become softer. They evolve from raised and tender nodules to a bruised appearance to complete resolution; these bruise-like evolution lesions are known as “erythema contusiformis” [2,3,6,69]. Frequently, lesions in different stages of the evolution can be seen in the same patient. Sometimes, residual hyperpigmentation may take weeks to resolve. New outcropping may continue to arise for up to 6 weeks [2,3]. As well as in the prodrome, also during the EN course, other symptoms may be associated as fever, weight loss, malaise, cough, arthralgia and/or arthritis, headaches, abdominal pain, lymphadenopathy, and diarrhea. Arthralgia, mostly of ankle and knee, is common and has been reported in up to 70% of adults, is less frequent in the pediatric series: Kakourou et al. found it in 15% of cases [7] and Hassink et al. in 41% [10] and may persist after the skin lesions have resolved. If pharyngitis accompanies the cutaneous lesions, the streptococcal infection can be easily supposed. Pulmonary hilar adenopathy may develop as part of hypersensitivity reaction, especially in sarcoidosis. A variant of EN is erythema nodosum migrans (ENM) that typically presents as unilateral, migratory, relatively painless, nodular lesions, localized laterally rather than anteriorly on the leg. The lesions of ENM are fewer in number and tend to persist longer than those of EN, to undergo centrifugal spread with central clearing, and may assume a yellowish or morphea-like appearance [70].

### 3.5. Assessment and Diagnosis

**Figure 1:**
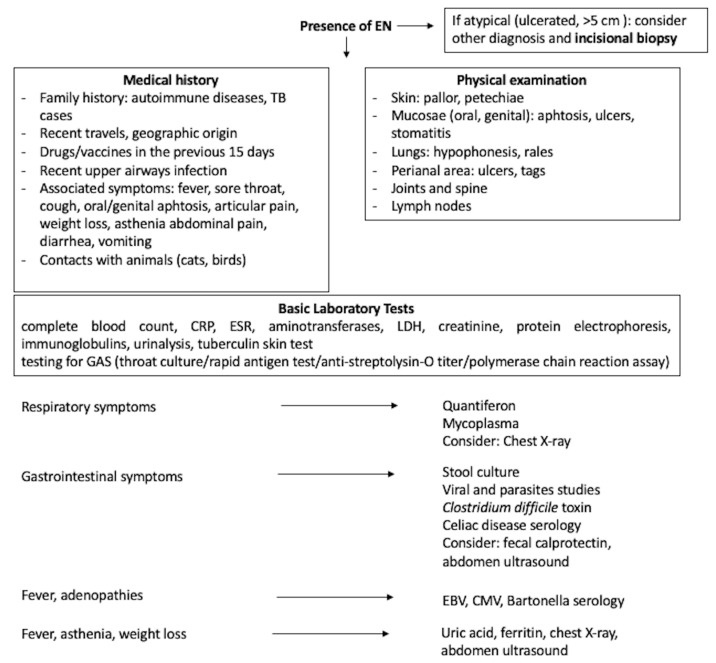
The diagnostic path facing children with EN. CMV: cytomegalovirus, CRP: C-reactive protein, EBV: Epstein–Barr virus, EN: erythema nodosum, ESR: erythrocyte sedimentation rate, GAS: Group A β-hemolytic *Streptococcus*, LDH: lactate dehydrogenase, TB: tuberculosis.

The diagnosis of EN is made on clinical grounds alone, due to the characteristic clinical features. When the nodules have an acute onset, are localized to the lower limbs, bilaterally distributed, and painful and red, EN should be the first choice, especially in children [2,71]. Biopsy is rarely required and should be performed only in patients with atypical presentation (ulcerated lesions or larger > 5 cm, or underlying immunosuppression). As already stated, the age of the patient and the EN location can guide in the clinical diagnosis. However, histopathology is essential for definitive diagnosis in uncertain cases, comprising recurrent or persistent EN. If required, a deep incisional biopsy is necessary to properly reach the subcutaneous tissue [1,65]. Pathology allows us to distinguish “mostly lobular” panniculitis (poststeroid, physical panniculitis) from “mostly septal” inflammation, the latter comprising EN [59]. In Figure 1, a schematic approach to the diagnostic pathway in children with EN is suggested. There are no routine specific laboratory tests for a child with EN; these should be “tailored” based on the history and physical examination. Therefore, these first two steps represent the source of the most useful information. The patient’s geographic location and travel history should be always considered to elucidate possible exposure to endemic infectious agents. The historical interview should also refer to the occurrence of constitutional symptoms (weight loss, fever, asthenia, loss of appetite), previous URTI, cough, dyspnea, sore throat, abdominal symptoms and bowel motions, and musculoskeletal complaints in the patient. The recent drug intake or vaccinations should be investigated considering the previous 15 days before EN onset. Regarding the family, the presence of autoimmune diseases and TB exposure should be thoroughly requested, and finally, the presence of animals in the home (cats or birds). The physical examinations should carefully evaluate skin (pallor or petechiae) and mucosa, in particular of the mouth (aphthous, ulcers, or stomatitis), genital and perineal areas (ulcer and tags), joint, spine, lymph nodes, lung, and abdomen, to promptly identify any further alteration. In any case, the possible underlying cause warrants some investigations. Among the basic laboratory tests complete blood count with differential, erythrocyte sedimentation rate (ESR), C-reactive protein level (CRP), or both, and urinalysis should be included. Leukocytosis with neutrophilia suggest a bacterial infection, atypical lymphocytosis an infectious mononucleosis, while eosinophilia is found in allergic disease or parasitic infestation; in contrast pancytopenia, or very marked leukocytosis suggests leukemia. Elevation of ESR is common in both idiopathic and secondary EN and was not discriminatory in children [7]; both ESR and CRP may be increased in many conditions (infectious, autoimmune diseases, and malignancy). Similarly, Dogan et al., in their study on 43 patients (adults and children) with EN, stated that ESR, CRP, and procalcitonin levels did not differ among secondary and idiopathic cases [72]. Serum aminotransferases, lactate dehydrogenase (LDH), creatinine, protein electrophoresis, and immunoglobulins should complete the routine blood workup. Testing for streptococcal infection (i.e., throat culture, rapid antigen test, anti-streptolysin-O titer, and polymerase chain reaction assay) should be required given the high incidence of GAS infection. Other infectious serologies should be selected considering the clinical manifestations and the microbiological infectious geographic prevalence (in our climates, hepatitis B virus, HCV, EBV, CMV, *Mycoplasma pneumoniae*, *Yersinia enterocolitica*, *Salmonella* spp.). QuantiFERON-TB, as well as tuberculin skin test (Mantoux), was strongly suggested particularly in case of exposure to TB [19]. Celiac screening should also be done, even without other symptoms/signs of CD. Patients with gastrointestinal symptoms should have a stool culture, research of viral or parasites and Clostridium difficile toxin on the stool; if indicated, fecal calprotectin and fecal occult blood research could provide useful information. Among imaging, the chest X-ray is still mandatory as it can reveal tuberculosis, other pulmonary infections, and lymphoma. Bilateral hilar lymphadenopathy suggests sarcoidosis, whereas unilateral hilar lymphadenopathy is more commonly seen in tuberculosis, brucellosis, and coccidioidomycosis [2,73]. Abdomen ultrasound could be helpful if gastrointestinal manifestations are present. Other investigations should be chosen case-by-case, for instance, a pregnancy test must be done in all sexually active adolescents, and angiotensin-converting enzyme (ACE) should be measured in the suspicion of sarcoidosis. Evaluating clinical and laboratory findings, Mert et al. reported that leukocytosis, elevated CRP and ESR, the presence of prolonged fever, cough, sore throat, diarrhea, arthritis, non-relapsing EN, and an abnormal chest X-ray were predictors of secondary EN, while recurrent EN predicted primary EN [6]. The differential diagnosis in children should include cellulitis, Henoch-Schönlein purpura, α1-antitrypsin deficiency, cold-induced panniculitis and cutaneous polyarteritis nodosa. Furthermore, the subcutaneous bacterial/mycobacterial/fungal infections can resemble EN. Finally, the battered child syndrome should be remembered. Superficial thrombophlebitis could be taken into account in the unusual case of unilateral EN. In adults, many other forms of panniculitis, including EN leprosum, erythema induratum of Bazin, pancreatic and lupus panniculitis, are included [16].

### 3.6. Prognosis

The prognosis of the skin lesions is excellent, with spontaneous resolution of the lesions in most patients within 2–6 weeks without scarring. However, if the antigenic stimulus persists, especially in the idiopathic form, the disease may last several months. Therefore, the clinician’s attention must be directed toward the presence of an underlying disease and treat it, as well as withdraw the drug, if identified as a trigger. A chronic form of the disease is also described, but it is quite rare [74]. Recurrent EN, although uncommon, has been reported not only in adults [6], but also in children [10]. Recurrences occur in about a third of cases, especially if EN is idiopathic or the underlying causes are not adequately treated [75].

### 3.7. Treatment

Treatment of EN should be aimed at the underlying etiology, if known. Although medications are less often implicated in pediatric cases of EN, careful review of all medications is essential. Treatment should be focused on symptomatic support and removal of the causative agent if present. Rest, elevation, and compression may help in symptoms’ relief. Drugs can be given to reduce symptom and hasten resolution. Non-steroidal anti-inflammatory drugs (NSADs), such as naproxen, ibuprofen or, more rarely, indomethacin, have also been shown to be of benefit; however, caution is requested in patients with IBD as they may trigger a flare-up or worsen an ongoing acute disease episode. Second-line agents for recurrent or chronic disease including colchicine, hydroxychloroquine, and dapsone are reported only in adults, mostly for chronic or recurrent cases [76,77]. More aggressive treatment also may be tailored to disease-specific regimens in case of secondary EN: steroids used in combination with hydroxychloroquine, cyclosporin A, or biologic agents (anti-interleukin 12/23, Vedolizumab) have been used to treat autoimmune disease-associated EN [78,79]. Intralesional and/or systemic corticosteroids may be considered for severe cases, and eventually used with caution after exclusion of infectious disease [69]; a dose of 0.5–2 mg/kg/day of prednisone for a period of 1–2 months had variable success in one pediatric case series [80].

## 4. Conclusions

EN is a self-limited autoimmune panniculitis. It may be idiopathic or associated with a wide variety of infectious and non-infectious diseases, including sarcoidosis, rheumatologic diseases, inflammatory bowel diseases, medications, autoimmune disorders, pregnancy, and malignancies, some of which are severe and life-threatening. Although it has no specific cause in about half of cases, it is imperative to investigate possible triggering causes; thus, an optimized cost-effective initial workup is necessary. Pertinent physical examination findings and historical clues often direct the pediatrician toward the appropriate diagnostic path selecting the adequate laboratory investigations and imaging studies of the initial workup. Such tests should be done in a stepwise fashion depending on clinical suspicion.

## Figures and Tables

**Table 1:** Etiology of EN in children [1,2,16].

Primary	Idiopathic		
**Secondary**	Infectious	Bacterial	
		Fungal	
		Viral	
		Protozoal	
	Systemic	Sarcoidosis	
		IBD: CD, UC	
		Behçet Disease	
		Sjogren Disease	
		Celiac Disease	
		Autoimmune Hepatitis	
		Spondiloarthropathy	
		Vasculitis: Kawasaki, Takayasu	
		Monogenic Autoinflammatory Diseases: Blau Syndrome, CAPS, HIDS	
		Sweet syndrome	
	Malignancy	Lymphoprolipherative: Leukemia, Lymphoma (Hodgkin and non-Hodgkin) Solid Tumors: Carcinoid, Sarcoma	
	Others	Drugs: Antibiotics (Penicillin, Macrolides, Cephalosporin), PPI, Everolimus, Aspirin, Contraceptives, Azathioprine, Phenitoin, Valproate	
		Vaccination: BCG, DTaP, HPV, SARS-CoV-2	

BCG: bacillus Calmette-Guérin, CAPS: cryopyrin-associated periodic syndrome, CD: Crohn’s disease, DTaP: diphtheria, tetanus, and acellular pertussis, EN: Erythema nodosum, HIDS: HyperIgD syndrome, HPV: human papillomavirus, IBD: inflammatory bowel disease, PPI: proton pump inhibitor, SARS-CoV-2: severe acute respiratory syndrome coronavirus 2, UC: ulcerative colitis.

**Table 2:** Etiologic and epidemiologic data extracted from the pediatric cases series.

	Labbé 1996 [5]	Kakourou 2001 [7]	Hassink 1997 [10]	Garty 2000 [14]	Aydın-Teke 2014 [11]	Litwin 2014 [15]	Picco 1999 [12]	Cengiz 2006 [13]
N° tot cases	27	35	36	24	39	12	22	10
M/F	15/12	17/18	18/18	8/16	18/21	5/7	12/10	5/5
Mean age, yrs.	9	8.9	10	9.9	11.3	11.9	10.4	8.8
Idiopathic, %	41	23	22	33	44	25	27.2	50
Secondary, %	59	77	78	67	56	75	72.7	50
Infectious, %	55	71	55	46	51	50	45.4	50
GAS	22	48	27	25	22	25	22.7	30
TB		5.7			7.5		4.5	20
RTI	11	8.5	13.8		5.5	8.3		
GI	22	8.5	8.3		2.5	8.3	4.5	
Cat scratch				4				
EBV			2.7	17			4.5	
CMV					2.5			
HPV-B19			2.7					
Tularemia					10.2			
Other						8.3		
Sarcoidosis	4		2.7		2.5			
IBD		2.8	16.6	13		25	13.6	
Behçet			2.7	8	2.5		9	
Malignancy		2.8						
SpA							9	

CMV: cytomegalovirus, EBV: Epstein–Barr virus, F: female, GAS: Group A β-hemolytic Streptococcus, GI: gastrointestinal, HPV-B19: human Parvovirus B19, IBD: inflammatory bowel disease M: male, RTI: respiratory tract infection, SpA: spondyloarthropathy, TB: tuberculosis, yrs.: year.

**Table 3:** Infectious causes of erythema nodosum in children [7,24,25].

Bacterial	Viral	Fungal	Protozoal
GAS	EBV	Candida albicans	Giardia lamblia
Mycobacterium tuberculosis	HBV	Trichophyton mentagrophytes	Entamoeba histolytica
Atypical mycobacteria	HCV	Coccidioides immitis	Toxoplasma gondii
Yersinia enterocolitica	HPV B19	Blastomices dermatitidis	
Salmonella spp.	HIV	Histoplasma capsulatum	
Campylobacter jejuni	CMV	Sporothrix schenckii	
Mycoplasma pneumoniae	Parapoxvirus		
Chlamydia trachomatis	VZV		
Chlamydia psittaci	SARS-CoV-2		
Coxiella burneti			
Bartonella henselae			
Helicobacter pylori			
Gardnerella vaginalis		y	
Francisella tularensis			
Leptospira			
Brucella spp.			
Shigella flexneri			
Burkholderia pseudomallei			

CMV: cytomegalovirus, EBV: Epstein–Barr virus, GAS: Group A β-hemolytic *Streptococcus*, HBV: hepatitis B virus, HCV: hepatitis C virus, HIV: human immunodeficiency virus, HPV-B19: human Parvovirus B19, SARS-CoV-2: severe acute respiratory syndrome coronavirus 2, VZV: varicella zoster virus.

## Data Availability

Not applicable.
